# Travels through Different Provinces of the Russian Empire

**Published:** 1781-01

**Authors:** 


II. P. S. Pallas, D. A. D. Profeſſors der Naturgeſchichte und ordentlichen Mit gliedes der Ruſſiſch-Kaiſerlichen Akademie der Wiſſenſchaften, der freyen öKonomiſchen Jeſellſchaft in St. Peterſburg, wie auch der Römiſch-Kaiſerlichen Akademie der Naturforſcher und Königlichen Engliſchen Societat; *Reiſe durch verſchiedener Provinzen des Ruſſiſchen Reichs*; i. e. *Travels through different Provinces of the Ruſſian Empire*, *by* P. S. Pallas,

Pallas, M. D. *Professor of Natural History and Member of the Imperial Academy at St. Petersburgh*, F. R. S. &c. 4to. Petersburg. Vol. I. 504 Pages, with eleven Copper Plates.

THE medical and philosophical reader cannot fail of being highly gratified by a perusal of the work before us: it consists of three large volumes in 4to. and is enriched with several very interesting engravings. We shall give an account of the first of these three volumes in our present number, and of the two others in our next, extracting from each of them such passages as seem to be the best suited to the nature of our plan.

Our ingenious author begins with describing his travels during the years 1768 and 1769 from St. Petersbugh through Moscow, Woldimer, Mordua, Samara, Orenburg, and Jurieu, towards the Caspian Sea, and from thence to Ufa, where he wintered.

At Waldai he observed great abundance of the *Gordius Aquaticus*, a species of worm that destroys fish by perforating their branchiæ. In the countries bordering on the lower part of the Wolga

Wolga this worm is faid to be frequently met with in ulcers in the human fpecies.

Near the convent of Nifkolfkoy, and in other parts of the government of Cafan, the Uva Urfi (by the Ruffians named *Toloknjanik*) grows in great plenty: it is much employed in tanning, the fkins being found to be much more fpeedily and better tanned by means of this plant.

In the river Mofkua the *fpongia fluviatilis*, Linn. occurs in great quantity. The women of the lower clafs dry and employ it as a cofmetic. The fmell which this plant yields in burning would feem to indicate that it is of an animal nature; but Dr. Pallas was not able to difcover the leaft appearance of irritability in it.

In the Botanic Garden at Mofcow there is a confiderable plantation of the *Rheum Palmatum*, the growth of which is promoted by frequent tranfplantations.

On the roots of the *Hypericum perforatum* there is found a coccinella, not inferior to that met with in Poland.

The milky juice of the *Elvela Acaulis*, is employed as a domeftic remedy againft fcrophulous tumours, and indurated fwellings of the legs in perfons advanced in life.

In the neighbourhood of Murom the juice of the *Euphorbia Paluſtris*, or the dried root, is uſed as a purge. It acts at the ſame time as a gentle puke, and is held in conſiderable repute as a remedy for agues, indurations of the abdominal viſcera, and other chronic diſeaſes.

Our author recommends the culture of the *Polygonum convolvulus*, inſtead of buck-wheat, as yielding a greater quantity of ſeed, and bearing the cold better.

The dry woolly leaves of the *Centaurea Siberica*, after being bruiſed, are uſed as an application to green wounds. The *Cineraria Paluſtris* bruiſed, and made into an ointment with oil, is likewiſe applied to ulcers.

The poor people collect a great quantity of *Fungi* for the winter, and uſe them, either dry or ſalted, as food. They are by no means nice in collecting them, employing even ſuch as are worm eaten, and rejecting only a very few ſpecies.

Dr. Pallas gives a very particular account of the manner of preparing the *Juſten* or Ruſſian leather. It is chiefly tanned, it ſeems, with the bark of *Salix Arenaria*, and is rendered ſoft and pliable, not, as hath been hitherto ſuppoſed, by by the *ſedum paluſtre*, but by means of the thinneſt and pureſt oil of *Betula* (birch-tree). The *Santalum* alone is employed to give it a red colour; or the *Santalum* and green vitriol, when a black colour is wanted.

The root of Mandragora grows in the neighbourhood of Arſamas, and is much uſed. Our author likewiſe found there the *Veratrum Album*, which proves poiſonous to ſheep, horſes, and poultry. The inhabitants apply the powder of this plant to ulcers in their cattle, and it is ſometimes mixed with honey, and taken as a remedy for the *tænia lata*. They hold the *gentiana campeſtris* in great eſtimation as a cure for the bite of a mad dog, Nothing however can be more ſuſpicious than the pretended efficacy of remedies of this ſort.

We are told that near the river Piana the *cantharides* live on the *lonicera Tartaria*.

Our author gives an accurate deſcription of the Aſphalt Spring on the river Sock. The ſurface of the water is covered with a black and viſcid *aſphaltum* of the colour and conſiſtence of tar. On removing this ſubſtance (which is renewed again in a few days) there appears under it a fine penetrating oleum petrolei. The inhabitants tants employ this impregnated water as a gargle and drink in aphthæ and ulcerations of the mouth and throat. They likewiſe apply the *aſphaltum* itſelf to freſh wounds, or make it with butter into an ointment for ulcers. Some of them take it internally, boiled in milk, in obſtinate colics and in venereal caſes. On the ſame river Mr. Pallas met with ſeveral copious ſulphureous ſprings, the waters of which are employed as a hot bath in the itch and other cutaneous diſorders. He here adds an exact account of a ſulphureous lake about ſixty fathom in length, and forty-five in breadth, which emits a viſible vapour, and a diſagreeable ſmell of hepar ſulphuris that extends to the diſtance of ſeveral miles.

Speaking of the preparation of iſinglaſs, our author informs us that it is procured from the air-bags of fiſhes. At Simbirſkon on the river Wolga the air-bag of the *accipenſer Sturio*, L. is eſteemed the beſt for this purpoſe, and next to this that of the *accipenſer Huſo.* The air-bag of the *accipenſer Stellatus*, P. is mixed with that of the accipenſer Sturio. The little bladders of the *accipenſer Ruthenus*, P. are ſaid to yield the moſt tenacious gluten of any. Theſe bags, when extracted from the fiſh, are firſt put into freſh water, and afterwards a little dried; their external coat is then removed, and the internal gliſtening one (which is the iſinglaſs) twiſted into various forms. The beſt is rolled up in the ſhape of ſmall crowns, the next compacted like a book, and the inferior ſort ſimply dried. Farther down the Wolga they boil theſe bladders in water, and pour the extracted gluten into a variety of ſhapes. On the river Okka, where the *accipenſer Ruthenus*, only occurs, the whole air-bag is employed for the ſame purpoſe by ſimply beating and drying it, without any other preparation. A ſimilar gluten is likewiſe obtained from the air-bags of the different ſpecies of the *Silurus* and *Barbus.*

At Jaizkoi the iſinglaſs obtained from the *accipenſer Huſo* is reckoned the worſt, and that from the *accipenſer Stellatus* the beſt.

Our author gives ſome account of the *caveak*, which is eſteemed as an aphrodiſiac. It is nothing more than the ſpawn of thoſe fiſhes. The beſt ſorts are procured from the accipenſer Sturio and accipenſer Stellatus.

Capſicum is much cultivated at Samara. The ripe pods are dried in a furnace, and ſold to the lower ſort of people, who uſe it as common ſpice. The coſmetic in the greateſt repute at Samara is the root of the *onoſma echioides* combined with oil.

Dr. Pallas deſcribes the *Glis Moſchatus*, which he diſtinguiſhes from the genus of beaver, and names *Sorex Moſchatus.* In this animal the ſmell of muſk, which is derived from glands ſituated under the ſquamous membrane of the tail, is of a much more penetrating nature than that of the common muſk.

Tarantulas, equal in ſize to thoſe of Italy, are to be met with in the environs of Samara. The bite of theſe inſects occaſions only a painful ſwelling.

The great mountain of ſulphur oppoſite to the mouth of the river Sock, conſiſts of a white, compact, and fine calcareous ſtone, in which are found great quantities of gypſum, combined with ſulphur.

At Samara the *Rumex Alpinus* is given to children, and likewiſe to cattle, as a remedy againſt worms. It is alſo uſed in dying.

At Tſcherkaſk a coccinella is collected from the roots of the *fragaria* and *potentilla reptans.* It is firſt ſeparated from the adhering earth by means of a ſieve, and then dried in a pan over a moderate fire, or in an oven.

Near Orenburg there are conſiderable mines of ſea ſalt, which is very pure, firm, ſolid, and white. The moſt common plant in that neighbourhood is the *Salicornia herbacea*, of which the inhabitants make an agreeable ſallad with ſpiced vinegar, as they do with other plants of the ſame kind. Speaking of the former plant, the Salicornia, our author remarks, that on account of the great quantity of common ſalt it contains, they are not able to prepare a ſoda from it.

The *Holcus ſaccharatus* is the only plant from which the Buchars prepare their bread. We are told that a ſingle plant often yields two pounds of ſeed.

A particular kind of leproſy is ſpoken of as beginning to make its appearance on the Jaik. At Aſtrachan it is called the diſorder of the Crimea. The firſt ſymptom of it is a blueneſs and ſwelling of the face: it goes on increaſing during four or five years, when it attains its higheſt degree of violence, and after ſeven years commonly proves fatal. All perſons are not equally affected affected by it. In the firft or fecond year painful fpots or flat tumours of a blueifh red colour appear on the fkin, and thefe are fucceeded by violent pains of the joints. When the fcurf dries away, the patient complains of an immoderate itching. It has often happened that when an unripe fcurf has been bruifed or imprudently opened, a fpreading ulceration has taken place, and the limbs have fallen off. The legs it feems are particularly affected with this diforder; and in five or fix years the cavities of the nofe, mouth, afpera arteria, and pharynx, are in a ftate of ulceration.

Speaking of the Kalmucks, our author obferves, that mares milk is greatly efteemed by them on account of its becoming fo fpirituous when four, that two or three large difhes of it are fufficient to intoxicate a man. This milk when frefh drawn is more fluid than cow's milk, but its peculiar flavour renders it fomewhat unpalatable. It lofes this tafte however as it turns four, and becomes more agreeable. In fummer it conftitutes almoft the only drink of the Kalmucks, and they diftil brandy from it. In order to turn it four, they pour it into large leathern bags or other veffels, which in winter they place near near the fire. In general the filthineſs of their veſſels is ſufficient for the purpoſe; but if not, they add a ferment of bread, or the remains of their ſour milk, or the coagulated milk they find in the ſtomachs of their lambs. Mr. Pallas accurately deſcribes the manner in which they obtain brandy from milk. Cows milk, we are told, does not yield quite a quarter, but from mares milk they are able to procure one-third part of indifferent brandy. That obtained from cows milk is ſeldom inflammable till it has been twice diſtilled.

Theſe people are not very delicate in the article of diet. They eat the meat of animals that die either a natural or violent death. As a ſubſtitute for tea, which they boil with milk and butter, they uſe the *glycyrrhiza aſpera.* They prepare their veſſels for culinary and other purpoſes from the hides of cattle.

The diſeaſe that is the moſt fatal to them is a contagious fever, which uſually carries off the patient on the eighth day. The itch and the lues venerea are likewiſe common diſorders among them, and they are in general ſubject to ophthalmia. This latter complaint is owing to the ſmoke of their cabbins and the heat of the the ſun. The ſmall pox makes great havock among them whenever it happens to be epidemic. Another tribe of Tartars, the Kirgis, are in ſuch dread of this diſeaſe, that as ſoon as it makes its appearance, they fly from the unhappy patients, leaving them only victuals and drink; and if a perſon with the ſmall-pox comes near their habitations, they immediately let fly their arrows at him.

The Kalmuck women are extremely hardy. They do all their ordinary buſineſs, and even get on horſeback, on the ſecond day after their delivery.

The Coſſacks employ the *ſerratula amara* as a cure for agues. This plant has a bitteriſh taſte like the centaureum. They likewiſe apply it powdered to ulcers in their cattle.

In Gurjef the tender leaves of the *rhaponticum* are taken in the ſpring as an antiſcorbutic. A decoction of the root is employed as a purge, and the Kirgis uſe it to dye their leather yellow. At Aſtrachan they prepare *capparides* from the *zygophyllum fabago*.

The Kalmucks are in great fear of the *phalangium arenoides*, a ſpecies of ſpider, and not without good reaſon, for its bite occaſions excruciating cruciating pain and violent ſwellings, which uſually prove fatal, unleſs ſweet oil, a remedy they ſucceſsfully employ in theſe caſes, is ſpeedily had recourſe to.

Dr. Pallas informs us, that the Tartars have a cuſtom of removing the hair from their bodies by means of a paſte made of nine parts of quick-lime and one of auripigmentum. We remember to have ſeen a formula of this kind in ſome of our old diſpenſatories, under the name of unguentum depilatorium.

In the ſpring a gummy ſubſtance exudes from the trunks of old larch trees, greatly reſembling gum Arabic, except in its being of a darker colour. This is a produce we ſhould not have expected from a ſpecies of tree that uſually yields only reſinous juices.

[ *To be continued.* ]

