# Errata

**Published:** 1781-06

**Authors:** 


					-p. 24, 1. 1, for
ledum read ledum.?p. 26, 1. 19, for caveat read cavear.-

				

